# Are Neoclassical Canons Valid for Southern Chinese Faces?

**DOI:** 10.1371/journal.pone.0052593

**Published:** 2012-12-28

**Authors:** Yasas S. N. Jayaratne, Curtis K. Deutsch, Colman P. J. McGrath, Roger A. Zwahlen

**Affiliations:** 1 Discipline of Oral and Maxillofacial Surgery, Faculty of Dentistry, The University of Hong Kong, Sai Ying Pun, Hong Kong; 2 Eunice Kennedy Shriver Center, University of Massachusetts Medical School, Waltham, Massachusetts, United States of America; 3 Department of Psychiatry, Harvard Medical School, Boston, Massachusetts, United States of America; 4 Discipline of Periodontology and Public Health, Faculty of Dentistry, The University of Hong Kong, Sai Ying Pun, Hong Kong; The University of Tennessee Health Science Center, United States of America

## Abstract

**Background:**

Proportions derived from neoclassical canons, initially described by Renaissance sculptors and painters, are still being employed as aesthetic guidelines during the clinical assessment of the facial morphology.

**Objective:**

1. to determine the applicability of neoclassical canons for Southern Chinese faces and 2. to explore gender differences in relation to the applicability of the neoclassical canons and their variants.

**Methodology:**

3-D photographs acquired from 103 young adults (51 males and 52 females) without facial dysmorphology were used to test applicability of four neoclassical canons. Standard anthropometric measurements that determine the facial canons were made on these 3-D images. The validity of the canons as well as their different variants were quantified.

**Principal Findings:**

The neoclassical cannons seldom applied to these individuals, and facial three-section and orbital canons did not apply at all. The orbitonasal canon was most frequently applicable, with a frequency of 19%. Significant sexual dimorphism was found relative to the prevalence of the variants of facial three-section and orbitonasal canons.

**Conclusion:**

The neoclassical canons did not appear to apply to our sample when rigorous quantitative measurements were employed. Thus, they should not be used as esthetic goals for craniofacial surgical interventions.

## Introduction

**Figure 1:**
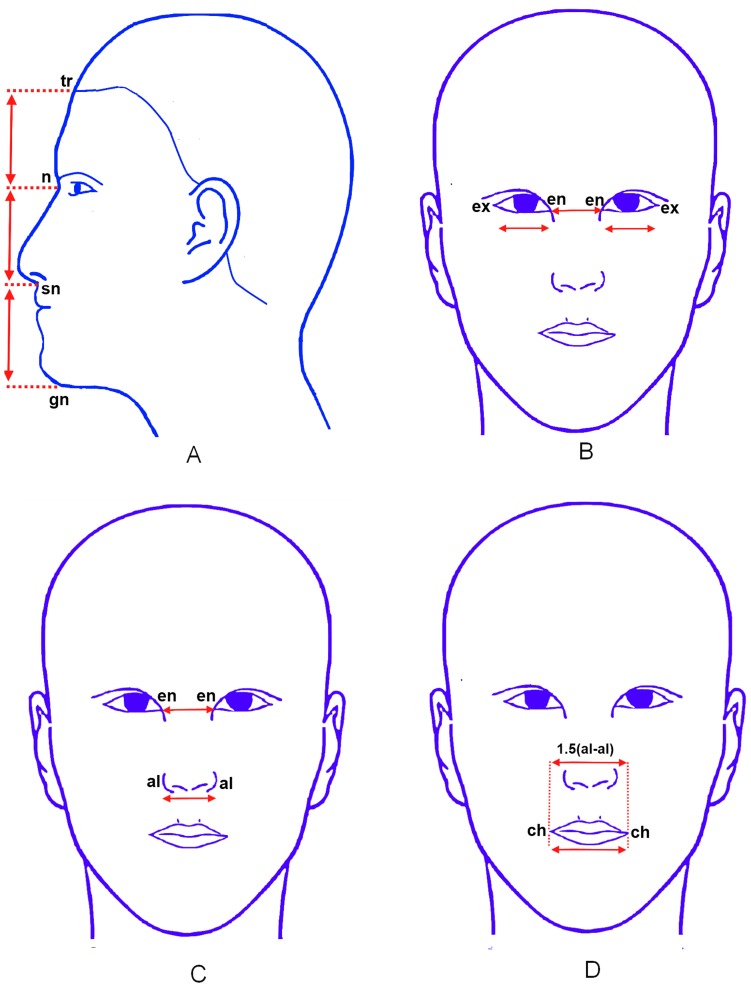
Some of the popular neoclassical canons. A - Facial three-section canon, B- Orbital canon, C-Orbitonasal canon, D- Naso-oral canon.

The human sculptures produced in ancient Greece, notably in the 4^th^ to 5^th^ centuries BC, were derived from proportions that followed established rules or ‘canons’. [1] These rules were incorporated to the “neoclassical canons” for the human face by Renaissance artists that included Leonardo da Vinci, Vitruvius, Bergmuller, and Dürer. [2], [3] These canons (Figure 1 and Table 1) were based on the assumption that certain fixed ratios existed between different parameters of a harmonious face. Subsequently, these canons were adapted by medical artists, anatomists and aesthetic surgeons and continue to be used to this day. [3]

**Table 1:** Some of the popular neoclassical canons.

Type	Name	Description	Equation*
Vertical canon	Facial Three-section canon	The face can be divided into equal thirds by horizontal lines passing through the eyes and mouth	tr-n = n-sn = sn-gn
Horizontal canons	Orbitonasal canon	The nose width equals the distance between the eyes	en-en = al-al
	Orbital canon	The distance between eyes equals the width of each eye	en-en = en-ex
	Naso-oral canon	The mouth width is one and one-half times the nose width	ch-ch = 1 ½ al-al

al – Alare, ch – Cheilion, en – Endocanthion, ex – Exocanthion, gn – Gnathion, n – Nasion, sn – Subnasale, tr – Trichion.

Farkas *et al* [3] were the first investigators to test the applicability of neoclassical facial canons, studying samples of 6, 12, and 18-year old North American Caucasians. Subsequently the applicability of these canons was also tested on several other ethnic groups including African-Americans [4], Turkish [5], Vietnamese [2], Thai [2] and Chinese individuals [2], [6]. These studies were performed with manual anthropometry in which measurements were directly obtained using anthropometric tools, e.g., spreading and sliding calipers. There are few studies [7], [8], [9] which have used two-dimensional (2-D) photographs to validate the applicability of these canons. However, use of such 2-D techniques for quantification of the 3-D morphology of the face has inherent methodologic limitations. [10]

Evaluation of facial aesthetics is a crucial to the planning of orthognathic surgery, facial plastic surgery, prosthodontic or orthodontic treatment. In these disciplines, a number of clinical textbooks and journal articles recommend derivatives of neoclassical canons as valid criteria that could be used during aesthetic evaluation. For example, the formulation of ‘facial thirds’ – in which the face is divided in the vertical plane in to three regions of equivalent height – is commonly used *in lieu* of the facial three-section canon. Moreover, the ‘rule of fifths’ [11] which divides the face in the transverse dimension to five equal parts, assumes that the intercanthal distance(which occupies the middle fifth) is equal to the nasal width and widths of the eyes. Therefore this rule encompasses orbitonasal and orbital canons.

**Figure 2:**
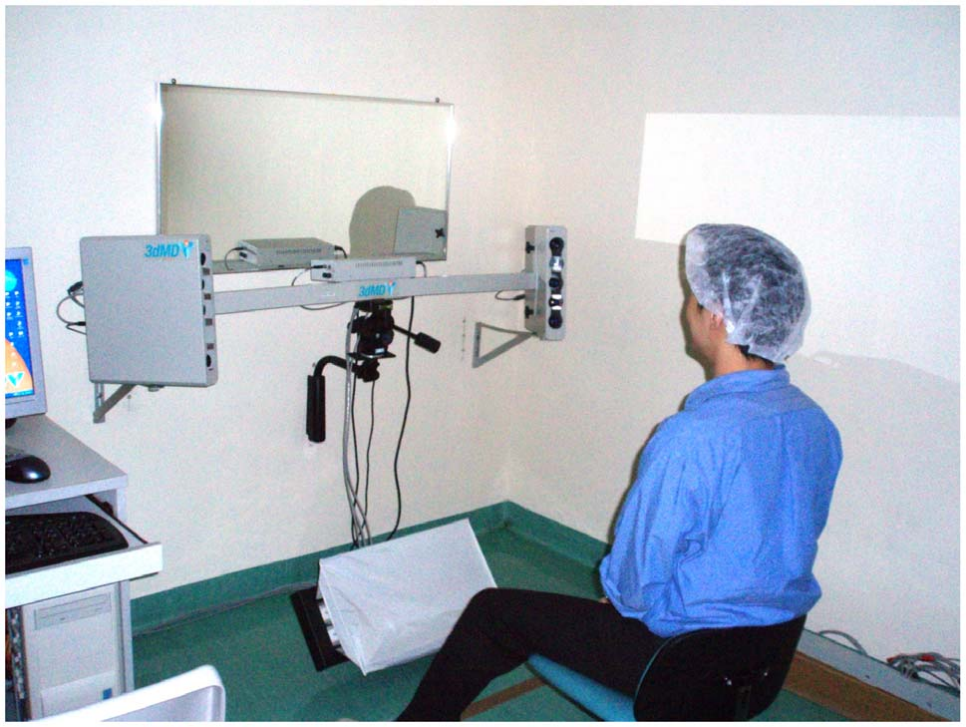
The stereophotographic system used for acquiring 3-D images.

How might one test these neoclassical formularies? With the advances in technology, non-invasive measurement systems based on stereophotography has been developed (Figure 2). Thus, it is now possible to perform anthropometric measurements on 3-D facial images, avoiding the need for direct contact with patients. Except for the work of Borman *et al* [5], previous studies on neoclassical cannons have used pooled samples of both genders and failed to explore the sexual dimorphism in relation to the prevalence of these canons. To overcome earlier methodologic limitations, the objectives of this study were to use stereophotography 1. to determine the applicability of neoclassical canons for Southern Chinese faces and 2. to explore the gender differences in relation to the frequency of occurrence of the neoclassical canons and their variants.

## Materials and Methods

The applicability of the following neoclassical canons for the Southern Chinese was tested;

- Three-section facial canon (tr-n = n-sn = sn-gn)
- Orbital canon (en-en = en-ex)
- Orbitonasal canon (en-en = al-al)
- Naso–oral canon (ch-ch  = 1 ½ al-al)

A detailed description of these canons can be found in Table 1. [3], [12], [13].

### Subjects

3-D photographs were acquired from 103 young adults (51 males and 52 females) from Hong Kong. All subjects that met the following inclusion criteria were used for this study.

- Ethnic Chinese
- Between 18–35 years of age
- No obvious facial deformities
- Class I skeletal pattern
- No history of maxillofacial or facial plastic surgery

### Ethics Statement

This study was approved by the Institutional Review Board of The University of Hong Kong/Hospital Authority Hong Kong West Cluster (Protocol No: UW 12-066). As no experimental interventions were performed on these subjects and due to the retrospective nature of this study a waiver of consent was granted by the IRB. The subject pictured here has granted his written consent as outlined in the PLoS consent form for publishing his photographs.

### Imaging Method

The *3dMDface* stereophotography system (3dMD, Atlanta, USA) was used to capture the 3-D facial photographs (Figure 2). Accuracy and reliability of this system has been previously validated. [14], [15] Subjects were imaged while sitting in a chair and looking at a mirror placed in front of them. A surgical cap was used to cover their hair but the hairline was kept slightly exposed.

### Image Analysis

**Figure 3:**
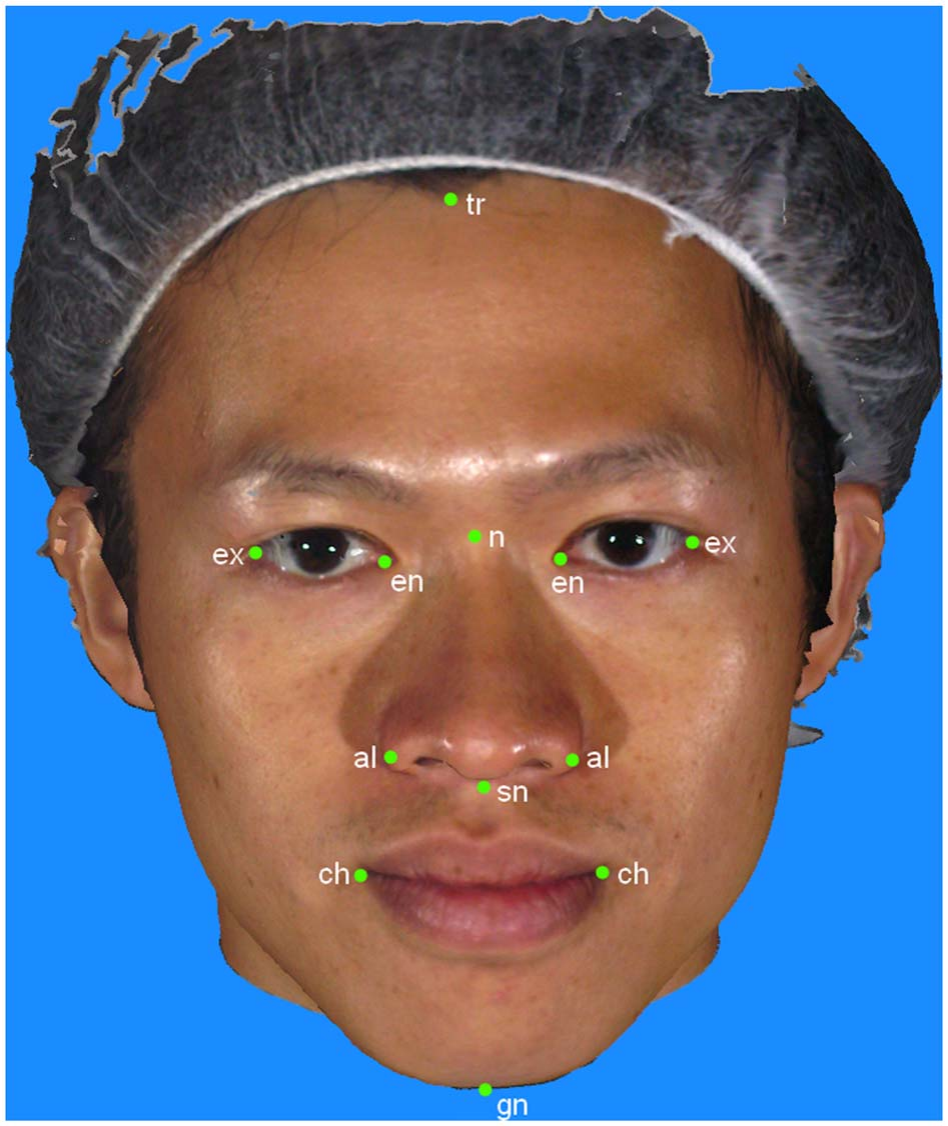
Anthropometric landmarks used in this study. al – Alare, ch – Cheilion, en – Endocanthion, ex – Exocanthion, gn – Gnathion, n – Nasion, sn – Subnasale, tr – Trichion,

The 3-D photographs were analyzed with the *3dMDVultus* software (Version 2.1, 3dMD, Atlanta, USA). Anthropometric landmarks that determine the facial Canons were selected on the 3-D images according to standard definitions (Figure 3 and Table 2). Landmark identification was performed by a single investigator (YSNJ) who had been trained by an expert in craniofacial anthropometry (CKD). Once the landmarks were identified on the 3-D photographs, a customized analysis template was created, and the software routine generated a spreadsheet containing inter-landmark distances.

**Table 2:** Definition of anthropometric landmarks used in this study.

Name	Abbreviation	Definition
Alare	al	The most lateral point on the nasal alar [23]
Cheilion	ch	The most lateral aspect of the vermilion border of the corner of the mouth [24]
Endocanthion	en	The inner corner of the eye fissure where the eyelids meet [23]
Exocanthion	ex	The outer corner of the eye fissure where the eyelids meet [23]
Gnathion	gn	The point on the inferior border of the mandible at which it intersects the midline [24]
Nasion	n	The midpoint on the soft tissue contour of the base of the nasal root [25]
Subnasale	sn	The midpoint of the angle at the columella base where lower border of the nasal septum and the surface of the upper lip meet [26]
Trichion	tr	A point at on the hairline in the midline of the forehead [26]

### Statistical Analysis

An *a priori* decision was made that a facial canon would be considered valid if the difference between the values predicted by the equations in Table 1 and the actual measurement was below 1.5 mm. Based on this threshold; the applicability of a canon for a particular subject was categorized as valid, smaller or larger. The mean en-ex measurement derived from right and left values were used when exploring the applicability of the orbital canon.

The frequencies of the valid canon and their different variants were calculated. A chi-square test was used to compare the prevalence of the different variants of neo classical facial canons between genders. These statistical tests were performed using IBM SPSS statistics version 20 (IBM, New York, NY, USA).

## Results

There were no statistically significant differences in relation to age between the male (24.2±3.03, range  = 20–32 years) and female (average  = 23.58±3.91, range  = 18–33 years) subjects (*p* = 0.372).

The application of the typical neo-classical canons was uncommon in this sample (Table 3). Notably, statistically significant sexual dimorphism was found in relation to the prevalence of the facial three-section (p = 0.005) and orbitonasal canons (p = 0.001).

**Table 3:** Prevalence of facial canons and their variants.

Canon	Canon and its variant	Male	Female	Whole sample	*P-value*
Facial three-section canon	tr-n = n-sn = sn-gn (%)	0	0	0	0.005
	tr-n>n-sn<sn-gn (%)	27.5	51.9	39.8	
	tr-n = sn-gn>n-sn (%)	13.7	17.3	15.5	
	sn-gn> tr-n>n-sn (%)	49.0	28.8	38.8	
	sn-gn> tr-n = n-sn (%)	2.0	1.9	1.9	
Naso–oral canon	ch-ch = 1 ½ (al-al) (%)	5.9	11.5	8.7	0.338
	ch-ch >1 ½ (al-al) (%)	2.0	5.8	3.9	
	ch-ch <1 ½ (al-al) (%)	92.2	82.7	87.4	
Orbital canon	en-en = ex-en (%)	0	0	0	–
	en-en>ex-en (%)	100.0	100.0	100.0	
	en-en<ex-en (%)	0	0	0	
Orbitonasal canon	en-en = al-al (%)	31.4	7.7	19.4	0.001
	en-en>al-al (%)	56.9	90.4	73.8	
	en-en<al-al (%)	11.8	1.9	6.8	

### Facial Three-section Canon

(tr-n = n-sn = sn-gn).

The typical facial three-section canon was not prevalent in this sample. The “forehead>upper face<lower face” type was more common among males (51.9%), the “lower face>forehead>upper face” type was more common among females (49%).

### Orbital Canon

(en-en = ex-en).

This canon was non-existent in this patient sample. All subjects had the en-en>ex-en variant confirming that the intercanthal distance was larger than the eye fissure length.

### Naso–oral Canon

(ch-ch  = 1 ½ al-al).

Only 8.7% of the subjects conformed to the naso–oral canon. The ch-ch <1 ½ (al-al) variant was the commonest indicating that mouth width was smaller in majority of males (92.2%) and females (82.7%) than predicted by the typical naso-oral cannon.

### Orbitonasal Canon

(en-en = al-al).

The orbitonasal canon was found in 19.4% of the combined sample and was more prevalent in males (31.4%). However, the en-en>al-al variant was common; it was more applicable in males (90.4%) than females (56.9%).

## Discussion

“Neoclassical canons” are frequently invoked (thought not by name) in current text books on orthodontics, prosthodontics, orthognathic surgery and plastic surgery, and they recommend these measurement prescriptions for the treatment planning. Despite the prevalence of their usage, these canons do not hold, as our project and other complementary studies [2], [3], [4], [6] have borne out. On the contrary, the overall applicability of the neoclassical cannons was low in our sample. For example, the facial three-section canon (tr-n = n-sn = sn-gn) and the orbital canon (en-en = ex-en) could not be found even in a single participant. The orbitonasal canon (en-en = al-al) was the most frequently supported, with a 19% prevalence in the whole sample. In summary, the typical neoclassical canons may not be applicable to the Southern Chinese faces.

Table 4 illustrates the comparison of our findings with those previously reported in the literature. [2], [3], [4], [6] Considerable variation in the applicability of these canons can be observed across different ethnic groups. The variant in the orbital canon with a wider intercanthal distance (en-en>en-ex) was found in 100% of the Hong Kong Chinese sample, remarkably higher than the 51.5% observed in North American Caucasians. The frequency of this variant in Southern Chinese were similar to Singapore Chinese. A relatively narrow-mouth with wide-nose variant of the naso-oral canon [ch-ch <1 ½ (al-al)] was common among all the East Asian ethnic groups and the African-Americans, whereas its converse variant [ch-ch >1 ½ (al-al)] was prevalent among North American Caucasian adults (60.2%).

**Table 4:** Overview of previous studies on neoclassical canons along with a comparison of findings in the current studyNR – Not reported.

Ethnic Group	N	Facial Three-section canon[tr-n = n-sn = sn-gn]		Orbital canon[en-en = ex-en]		Orbitonasal canon[en-en = al-al]		Naso-Oral canon[ch-ch = 1 ½ (al-al)]	
		Validity	Comments variation	Validity	Comments variation	Validity	Comments variation	Validity	Comments variation
North American White [4]	103	0%	tr-n>n-sn = 100%, n-sn<sn-gn = 100%	33.0%	en-en>ex-en = 51.5%	40.8%	en-en<al-al = 37.9%	20.4%	ch–ch >1 ½ al-al = 60.2%
Afro-Americans [4]	100	0%	tr-n>n–sn = 100%, n-sn<sn-gn = 95%	13.0%	en-en>ex-en = 73%	3.0%	en-en<al-al = 94%	1.0%	ch–ch <1 ½ al-al = 94%
Vietnamese [2]	60	0%	NR	0%	en-en>ex-en = 100%	16.7%	en-en<al-al = 76.7%	0%	ch–ch <1½ al-al = 100.0%
Thai [2]	60	0%	NR	0%	en-en>ex-en = 98.3%	21.7%	en-en<al-al = 76.7%	1.7%	ch–ch <1½ al-al = 98.3%
Singapore Chinese [2]	60	0%	NR	0%	en-en>ex-en = 100%	26.7%	en-en<al-al = 48.3%	1.7%	ch–ch <1½ al-al = 96.7%
Central Chinese [6]	206	NA	NR	35.5%	en-en>ex-en = 42.7%	35.4%	en-en<al-al = 34.5%	21.8%	ch–ch <1½ al-al = 71.8%
Southern Chinese	103	0%	tr-n>n-sn<sn-gn = 39.8%	0%	en-en>ex-en = 100%	19.4%	en-en>al-al = 73.8%	8.7%	ch-ch <1 ½ (al-al) = 87.4%

Many differences could be observed even among ethnic Chinese groups itself in relation to the facial canons. The applicability of the orbital and naso-oral canon was much higher in the sample from Mainland China reported by Wang *et al.* [6] compared to Singapore and Hong Kong Chinese. Even though this study mentions that the subjects were residing in central China, the authors have not specified the provinces from which they were recruited. Thus, it is difficult to make further interpretations about the origin of such differences. In addition, the en-en>al-al variant of the orbitonasal canon was more prevalent among the Southern Chinese (73.8%), whereas its converse variant (en-en<al-al) was common among the Central Mainland Chinese (34.5%) and Singapore Chinese (48.3%).

Further, we found significant gender differences in relation to the frequency of facial three-section and orbitonasal canon. Most of the earlier studies on neoclassical canons did not explore sexual dimorphism in relation to their occurrence as they pooled results from both males and females.

A complex assortment of main effects and interactions among genetic and environmental factors may have played significant roles in the genesis of morphological differences among ethnic groups. [16] Evolutionary forces such as founder effect or genetic drift resulting reproductive isolation and reduced genetic diversity at some time in that population’s history may have lead to the ethnic differences in the facial appearance. In addition, through sexual selection, individuals with attractive features may have been more likely to reproduce and pass on such traits to subsequent generations. [17] The dentition and associated masticatory musculature may have undergone changes as a consequence of differences in type of food consumed by these isolated populations. [18], [19] Thus, the size and shape of these muscles as well as protuberances in the facial skeleton required for their attachment would have been influenced by the diet. The variation in the nasal morphology along with the degree and distribution of subcutaneous fat may be a result of adapting to cold environments. [20], [21] These types of factors might have lead to significant ethnic differences in the facial morphology, e.g., of the types documented in the literature on anthropometric canons.

It was not possible to check the applicability of all the neoclassical canons^5^ cited in the literature due to some of the inherent limitations of landmark identification via stereophotogrammetry. The facial two-section, four-section and naso-aural canons were not tested as the vertex, zygion and some of the auricular landmarks cannot be identified accurately in stereophotographic images.

The aesthetic guidelines employed by present-day clinicians are rooted in the canons described for Renaissance art and sculptures, though to some extent they have been modified from the original. [22] However, based on the findings presented here, these canons do not hold for this Southern Chinese sample. Thus, rather than aiming to restore ideal facial proportions derived from the neoclassical canons, it would be prudent to make an objective assessment of facial aesthetics based on ethnicity and gender specific anthropometric norms.

### Conclusion

The anthropometric neo-classical canons for the most part did not apply to the Southern Chinese sample in this study. Thus, these canons do not provide useful formularies for planning surgical or non-surgical treatments for craniofacial dysmorphology.
